# Supplementary material for: ATP and Tri-Polyphosphate (TPP) Suppress Protein Aggregate Growth by a Supercharging Mechanism
Source: Biomedicines. 2021 Nov 9;9(11):1646. doi: 10.3390/biomedicines9111646 (PMC8616003; doi:10.3390/biomedicines9111646)
Supplement: Supplementary file 1 [file biomedicines-09-01646-s001.zip › biomedicines-1437166-supplementary.pdf]

Supplementary Information for

ATP and tri-polyphosphate (TPP) suppress protein aggregate growth by a supercharging mechanism

Figure S1A–D

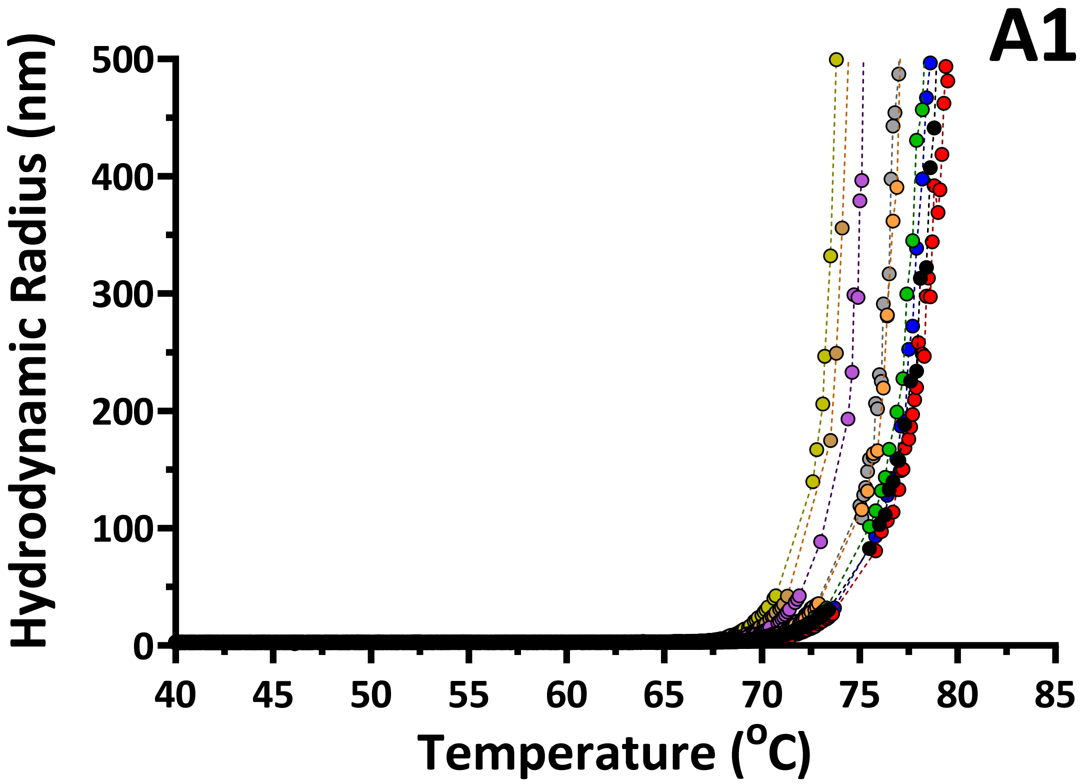

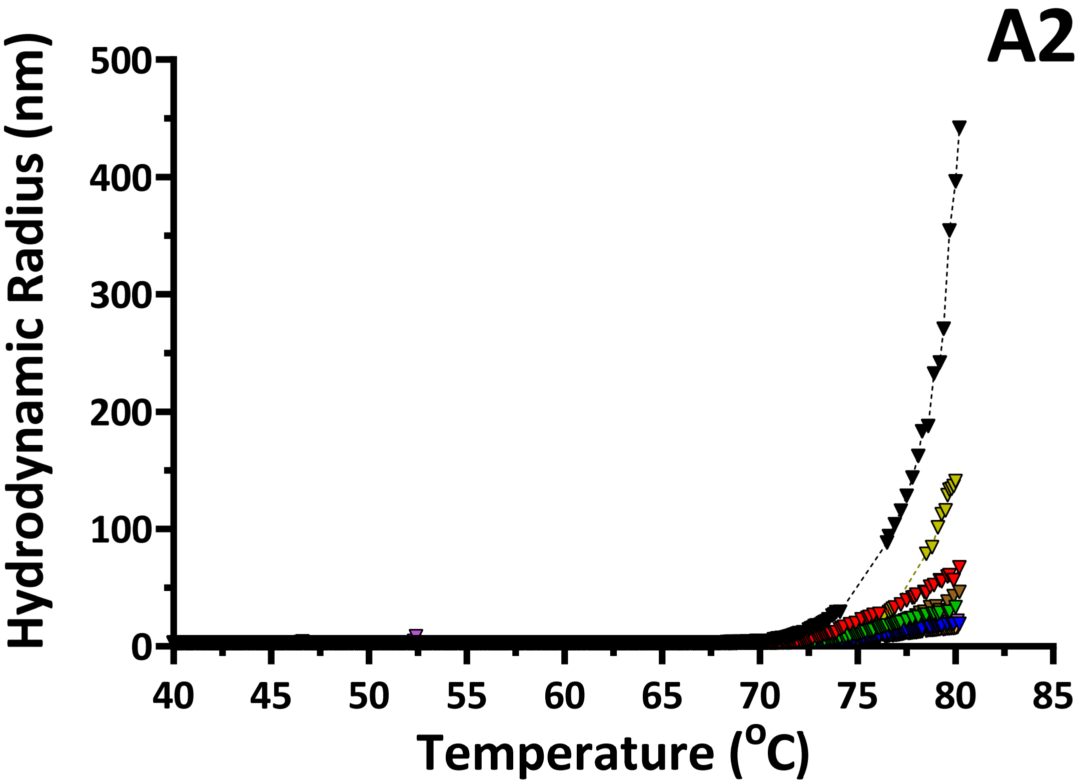

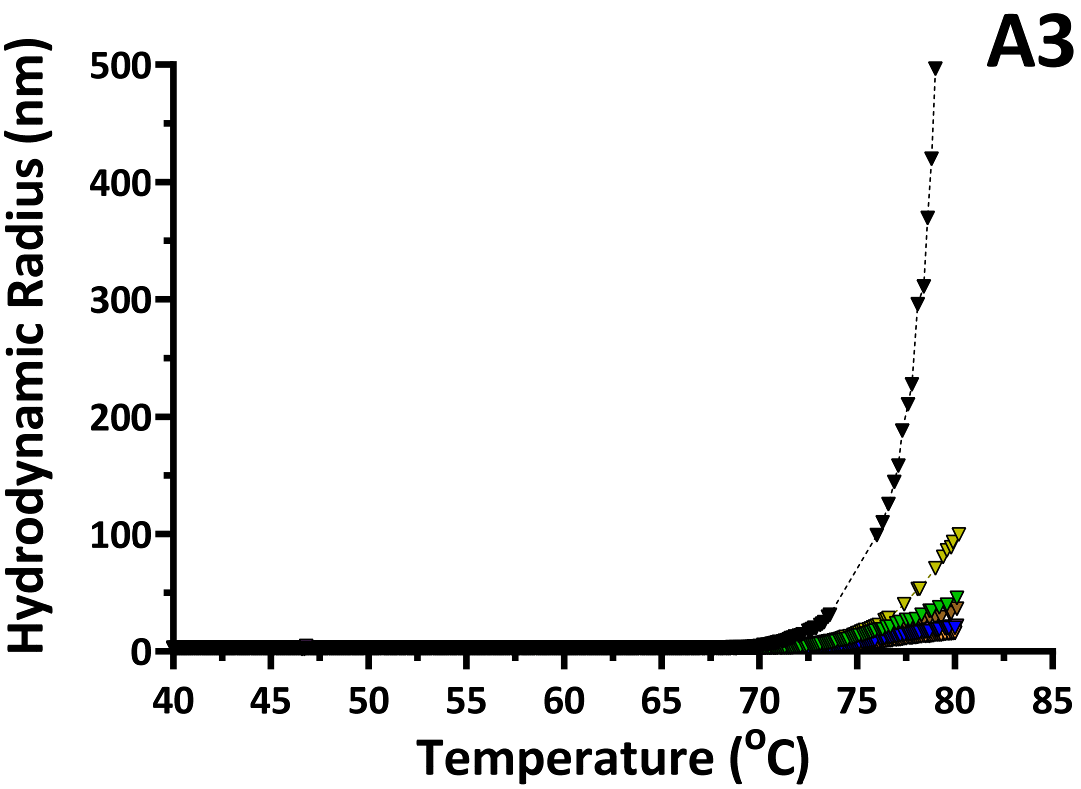

Figures S1: A1 to **A3**. Data for ovalbumin thermal ramped aggregation experiments with different concentrations of NaCl (**A1**), STPP (**A2**) and ATP (**A3**) in 10 mM Tris pH 7.0 buffer. NaCl (A1) was tested at 0 mM (●), 0.5 mM (●), 1 mM (●), 2.5 mM (●), 5 mM (●), 10 mM (●), 25 mM (●), 50 mM (●) and 100 mM (●). STPP (A2) was tested at 0 mM (▼), 0.5 mM (▼), 1 mM (▼), 2.5 mM (▼), 5 mM (▼), 10 mM (▼), 25 mM (▼), 50 mM (▼) and 100 mM (▼). ATP (A3) was tested at 0 mM (■), 1 mM (■), 2.5 mM (■), 5 mM (■), 10 mM (■), 25 mM (■), 50 mM (■) and 100 mM (■). It should be noted that not all data sets are clearly visible for STPP and ATP due to significant overlap. The dashed lines have been plotted to guide the eye and do not represent data or data fitting.

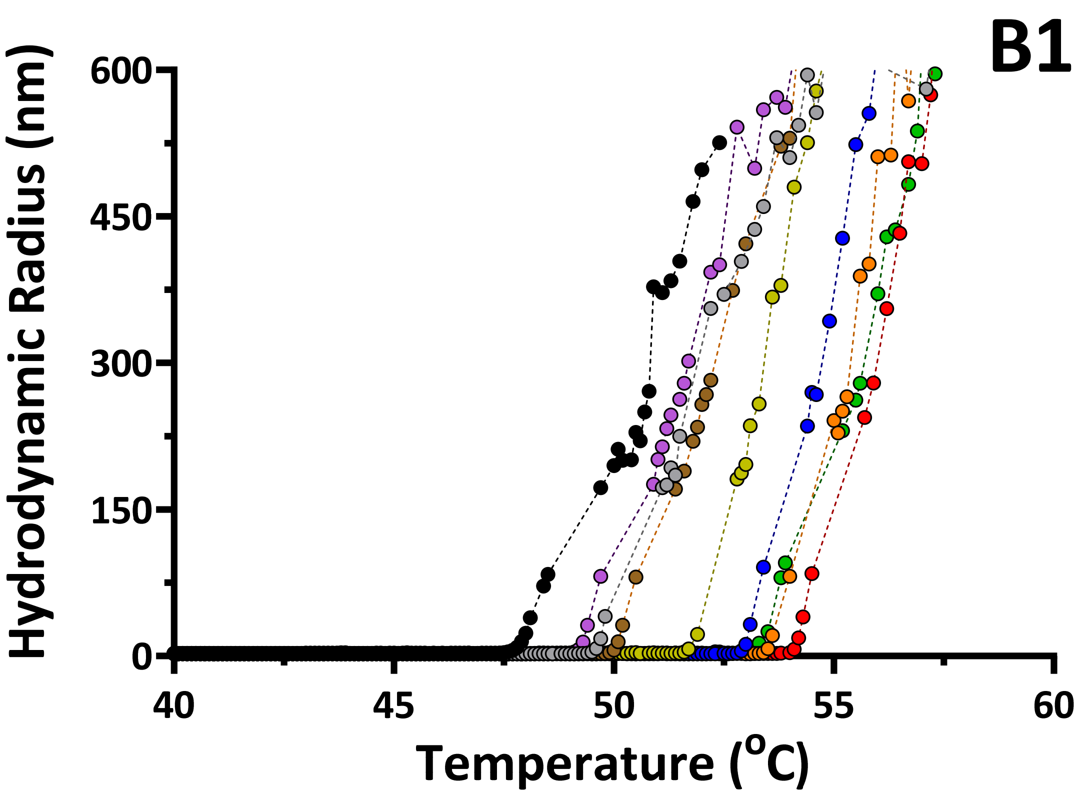
B1
Hydrodynamic Radius (nm)
600
450
300
150
0
40
45
50
55
60
Temperature (°C)

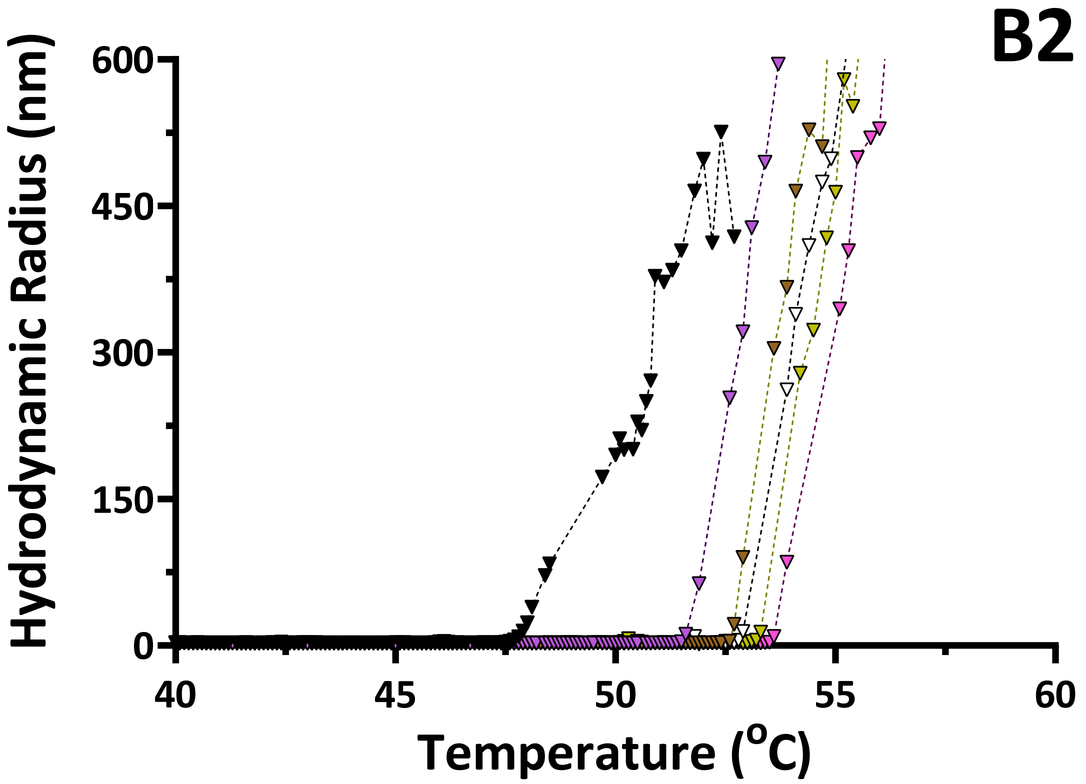
B2
Hydrodynamic Radius (nm)
600
450
300
150
0
40
45
50
55
60
Temperature (°C)

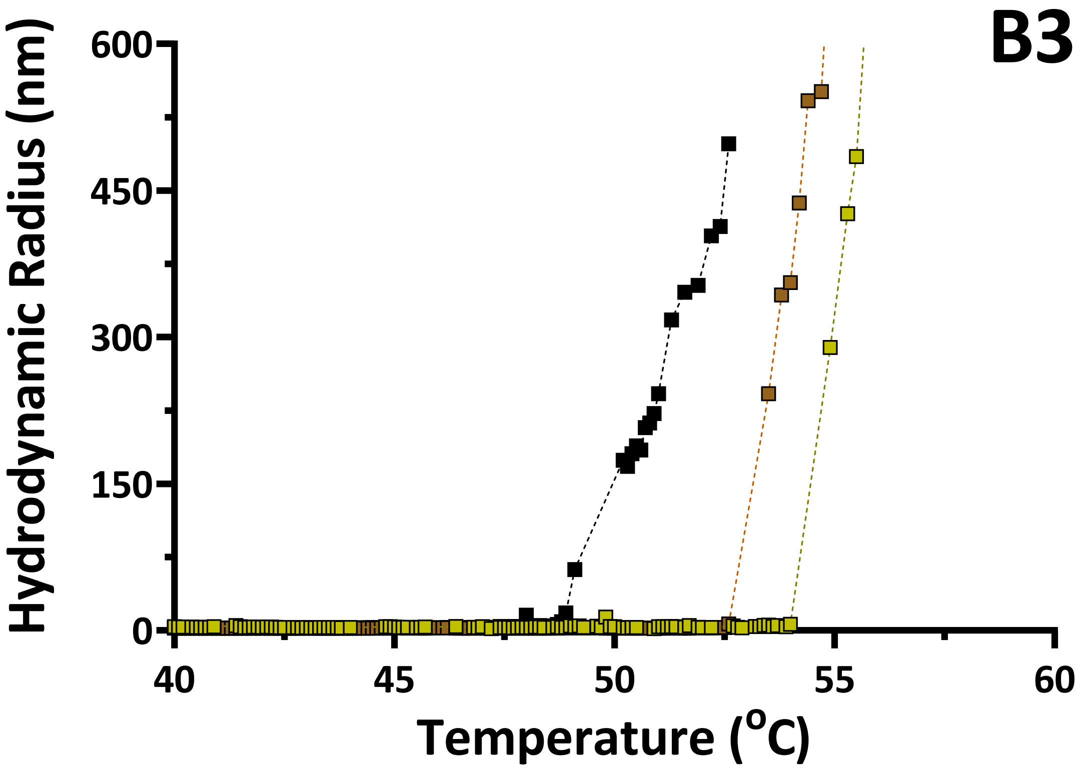

Figures S1: B1 to B3. Data for α-Cgn thermal ramped aggregation experiments with different concentrations of NaCl (**B1**), STPP (**B2**) and ATP (**B3**) in 10 mM Tris pH 7.0 buffer. NaCl (**B1**) was tested at 0 mM (●), 10 mM (●), 25 mM (●), 50 mM (●), 100 mM (●), 250 mM (●), 500 mM (●), 750 mM (●) and 1000 mM (●). STPP (**B2**) was tested at 0 mM (▼), 25 mM (▼), 50 mM (▼), 75 mM (▽), 100 mM (▼) and 150 mM (▼). ATP (**B3**) was tested at 0 mM (■), 50 mM (■) and 100 mM (■). It should be noted that not all data sets are clearly visible for STPP and ATP due to some overlap. The dashed lines have been plotted to guide the eye and do not represent data or data fitting.

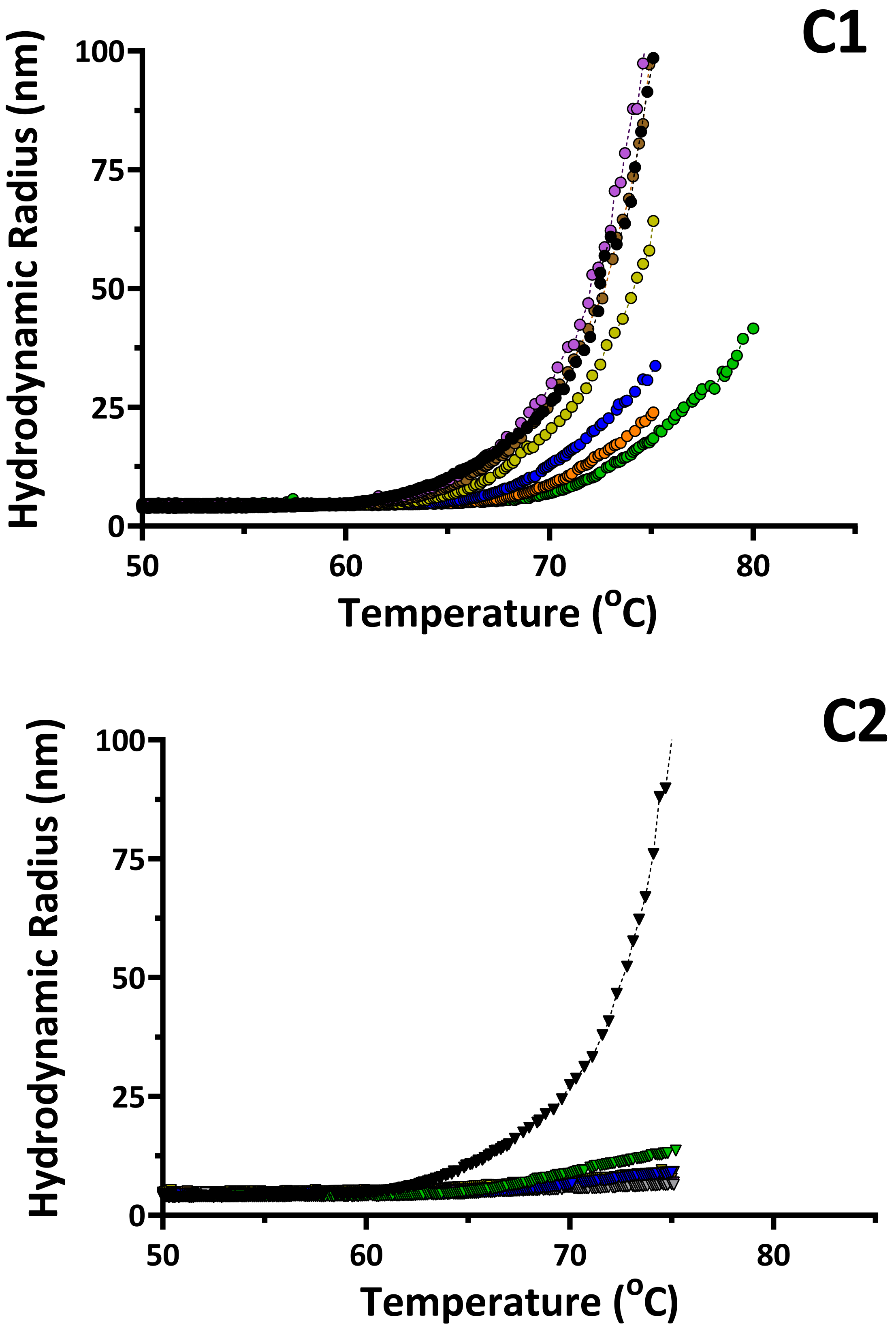

Figures S1: C1 to C2. Data for BSA thermal ramped aggregation experiments with different concentrations of NaCl (**C1**) and STPP (**C2**) in 10 mM Tris pH 7.0 buffer. NaCl (**C1**) was tested at 0 mM (●), 25 mM (●), 50 mM (●), 100 mM (●), 250 mM (●), 500 mM (●) and 1000 mM (●). STPP (**C2**) was tested at 0 mM (▼), 1 mM (▼), 2.5 mM (▼), 5 mM (▼), 10 mM (▼), 25 mM (▼), 50 mM (▼) and 100 mM (▼). It should be noted that not all data sets are clearly visible for NaCl and STPP due to significant overlap. The dashed lines have been plotted to guide the eye and do not represent data or data fitting.

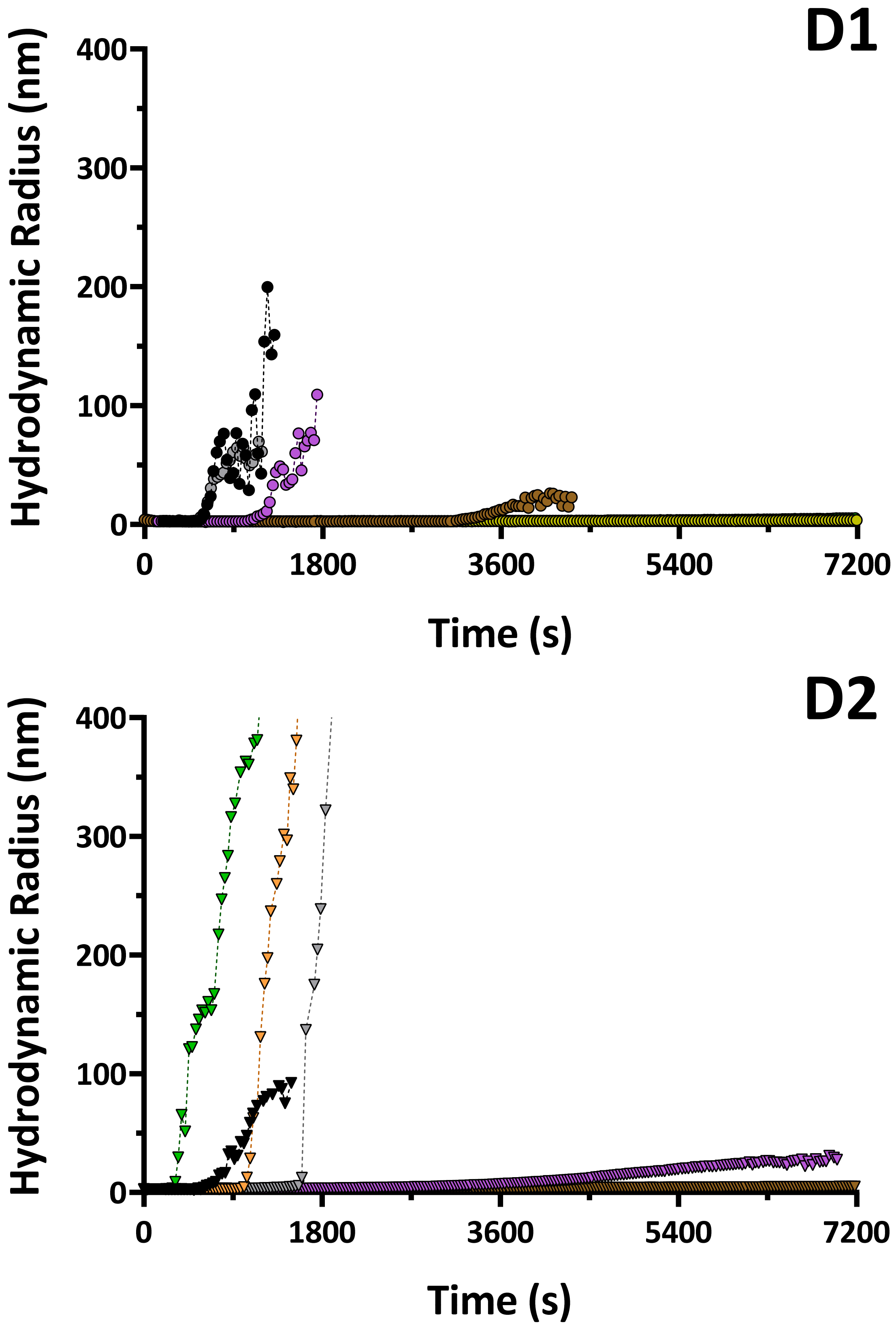

Figures S1: D1 to D2. Data for RNaseA thermal ramped aggregation experiments with different concentrations of NaCl (**D1**) and STPP (**D2**) in 10 mM Tris pH 7.0 buffer. NaCl (**D1**) was tested at 0 mM (●), 10 mM (●), 25 mM (●), 50 mM (●), 100 mM (●) and 1000 mM (●). STPP (**D2**) was tested at 0 mM (▼), 1 mM (▼), 5 mM (▼), 10 mM (▼), 25 mM (▼) and 50 mM (▼). It should be noted that not all data sets are clearly visible for NaCl and STPP due to significant overlap. The dashed lines have been plotted to guide the eye and do not represent data or data fitting.

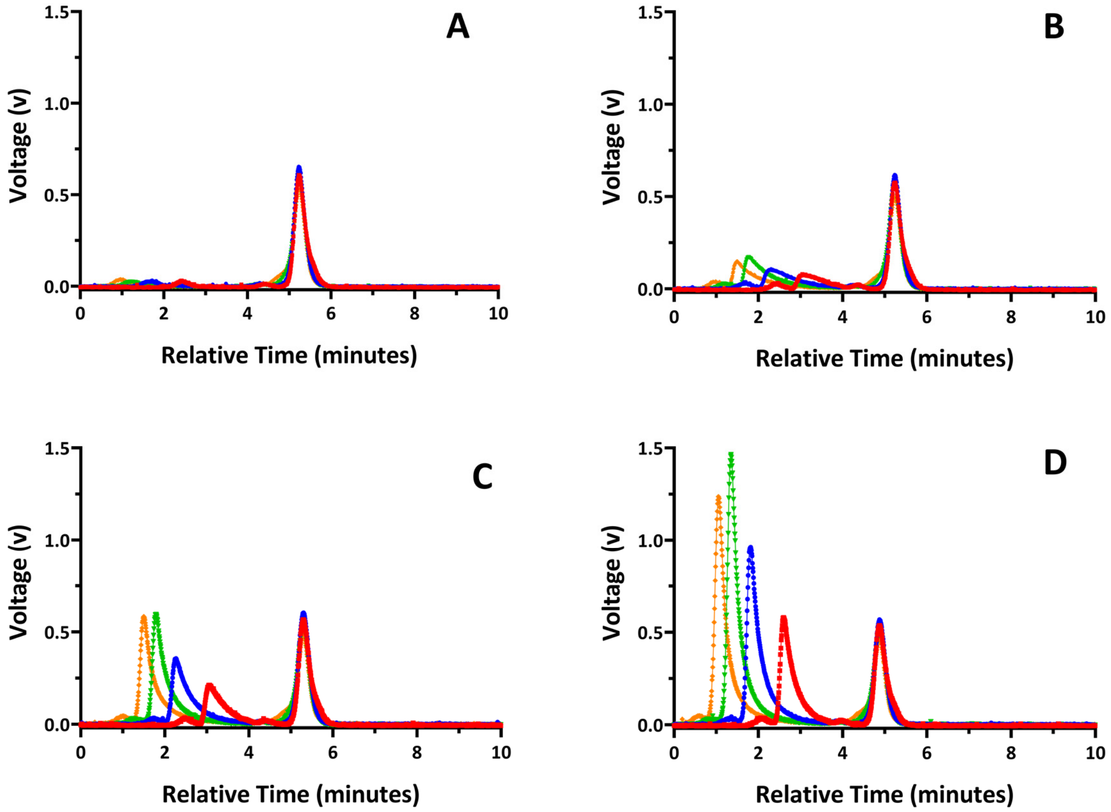

Figure S2. SEC-MALLS data for 1 mg/mL ovalbumin 10 mM tris pH 7.0 in the presence of 10 mM (red), 25 mM (blue), 50 mM (green) and 100 mM (orange) STPP overlays after heating at 70 ^{o}C for 0 minutes (A), 10 minutes (B), 20 minutes (C) and 30 minutes (D). Note. Relative times have been used for the x-axis on these graphs as they are overlays where corresponding monomer and aggregate pairs eluted from the column at different times throughout the experiment compared to each other. The purpose of these plots is to provide insight as to how the monomers and aggregates are eluted from the column relative to each other and the time scale over which they eluted from the column. The data presented here is from the detector at 90° relative to the laser beam.

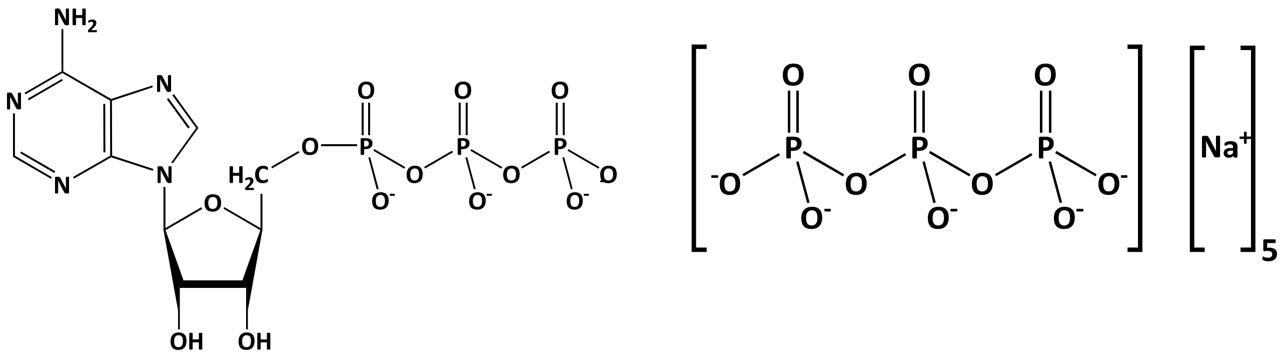

Figure S3. Structures for adenosine triphosphate (ATP) anion (left) pKa$_1$/pKa$_2$/pKa$_3$: <2, pKa$_4$: 6.48 and tripolyphosphate (STPP) anion (right) pKa$_1$: 1, pKa$_2$: 2.2, pKa$_3$: 2.3, pKa$_4$: 5.7, pKa$_5$: 8.5.
